# A Novel Approach to Monitor Clearance of Host Cell Proteins Associated With Monoclonal Antibodies

**DOI:** 10.1002/btpr.1948

**Published:** 2014-07-26

**Authors:** Nabila Aboulaich, Wai Keen Chung, Jenny Heidbrink Thompson, Christopher Larkin, David Robbins, Min Zhu

**Affiliations:** Bioprocess Development, Medimmune LLCOne Medimmune Way, Gaithersburg, MD, 20878

**Keywords:** host cell protein impurities, mAb process development, host cell protein clearance, proteomics, protein–protein interaction

## Abstract

Co-purification of a subset of host cell proteins (HCPs) with monoclonal antibodies (mAbs) during the capture of mAbs on Protein A affinity chromatography is primarily caused by interactions of HCPs with the mAbs. To date, there is limited information about the identity of those HCPs due to the difficulty in detecting low abundance HCPs in the presence of a large amount of the mAb. Here, an approach is presented that allows identification of HCPs that specifically associate with the mAb, while avoiding interference from the mAb itself. This approach involves immobilization of purified mAb onto chromatography resin via cross-linking, followed by incubation with HCPs obtained from supernatant of non-mAb producer cells that are representative of the expression systems used in mAb manufacturing. The HCPs that bind to the mAb are recovered and identified using mass spectrometry. This approach has not only allowed a comprehensive comparison of HCP subpopulations that associate with different mAbs, but also enabled monitoring of the effects of a variety of wash modifiers on the dissociation of individual HCP–mAb interactions. The dissociation of HCPs that associated with the mAb was monitored by enzyme-linked immunosorbent assay and mass spectrometry. This approach can be utilized as a screening tool to assist the development of effective and targeted wash steps in Protein A chromatography that ensures not only reduction of HCP levels copurified with the mAb but also removal of specific HCPs that may have a potential impact on mAb structural stability and patient safety. © 2014 American Institute of Chemical Engineers *Biotechnol. Prog*., 30:1114–1124, 2014

## Introduction

Host cell proteins (HCPs) originating from the production cell line are an important class of process-related impurities in biopharmaceutical products such as monoclonal antibodies (mAbs). Although HCPs are reduced to very low levels, typically <100 ppm, in the final mAb product, remaining HCP species may potentially induce immune response in patients by triggering formation of anti-HCP antibodies.^1^ It has also been reported that low levels of HCPs were observed to affect binding potency of a pure mAb product through blocking the epitope site of the mAb.^2^ In addition, HCPs can potentially affect product quality and stability through formation of undesired product variants, due to enzymatic activities of individual HCP species such as protease and disulfide reductase.^3^–^5^

The composition and abundance of HCP species remaining in the purified mAb product depend on many factors. The cell culture process as well as mAb harvest (primary recovery) conditions can potentially have a large impact.^6^,^7^ The distribution of HCPs co-purified with a mAb is also dependent on the types of chromatography techniques used in the manufacturing process; HCPs that have similar binding properties to those of the product may co-purified. In addition, the physicochemical properties of a mAb such as surface charges, hydrophobicity, and higher order structures may result in carryover of a subset of HCPs through nonspecific interaction with the mAb itself during the purification process.^8^,^9^ Some HCP impurities can potentially bind covalently to the mAb.

Protein A affinity chromatography is being used in the manufacture of biopharmaceuticals containing an Fc domain (including mAbs) because of its high specificity and ability to remove a large portion of HCP impurities.^10^,^11^ However, co-purification of HCPs with the mAb may occur even over Protein A chromatography, due to non-specific interactions of HCPs with the Protein A resin or with the mAb product molecule. The degree of HCP clearance depends partly on the backbone chemistry of the resin used.^8^,^12^ For example, higher levels of HCPs co-purify with the mAb on glass-based affinity resins compared to agarose-based resins, which has been ascribed to greater non-specific interaction of the HCPs with the glass backbone.^8^,^12^ Consequently, different wash buffers have been developed to reduce the levels of nonspecific interactions of HCP with the glass-based resin.^13^ Agarose-based affinity chromatography resins, on the other hand, show minimal non-specific interactions with HCPs. Accordingly, HCPs that co-elute with the mAb product on agarose-based resins do so predominantly through association with the product itself.^8^,^9^,^12^ A comprehensive study of HCP–mAb interactions has not yet been conducted. This is partially due to the technical challenges of identifying HCP species that associate with the mAb, as they are masked by the large amount of the mAb. Previous approaches to enhance detection and identification of HCPs included depleting the mAb using an orthogonal affinity resin, such as Protein G affinity.^14^ However, this approach requires careful optimization of the wash conditions to ensure complete HCP recovery without distorting the distribution of HCP species. An additional chromatographic step needs also to be incorporated to remove light chain fragments of the mAb, which may lead to unintended removal of some of HCP. Another approach that utilizes heterogeneous hexapeptide library beads provides a promising alternative. By limiting the retention of high abundance proteins relative to low abundance species, concentration ratios are normalized such that more HCPs can be identified.^15^

Levy et al.^16^ presented an approach to identify HCPs that interact with mAbs utilizing immobilization of the mAb onto a resin surface, followed by loading HCPs from null cell culture supernatant, wash, and elution of HCPs that associated with the immobilized mAb. As acknowledged by the authors, direct identification of HCPs was not possible due to incomplete elution of interacting HCPs, which led to insufficient detection using two-dimensional electrophoresis (2-DE). Interacting HCPs were instead identified using 2-DE to compare HCPs in the flow-through, containing non-interacting HCPs, to those in the null supernatant, containing all HCPs. HCP spots that were detected in the null supernatant and absent in the flow-through were assumed to be interacting with the mAb.^16^

Here we present a similar approach to identify HCPs that interact with the mAb without interference from the mAb itself. Covalent cross-linking of the mAb onto a resin surface was employed to immobilize the mAb. This was followed by loading HCPs from a null cell supernatant, wash, and elution using stringent conditions to recover HCPs that associated with the mAb. As the mAb remained bound to the resin during the elution of HCPs, our approach enabled a comprehensive proteomic analysis of recovered HCPs without mAb interference. In addition, our approach was utilized during screening of different wash conditions to dissociate HCP–mAb interactions and also to monitor the levels of individual HCPs that remained associated with the mAb after a particular wash. This work provides a more detailed understanding of HCP clearance, which has the potential to support the design of a rapid and targeted development of wash steps during mAb purification processes.

## Materials and Methods

### Materials

The mAbs used in this study, identified here as mAb1, mAb2, mAb3, and mAb4, have pIs of 9.4, 9.2, 8.1, and 8.7, respectively. The antibodies mAb1, mAb2, and mAb3 are immunoglobulin (Ig) G1 molecules, while mAb4 is an IgG2. These mAbs were purified at MedImmune (Gaithersburg, MD) and HCP levels were below 20 ppm as measured using custom enzyme-linked immunosorbent assay (ELISA) assay described below. HCPs were obtained from null CHO cells that were grown under similar conditions to mAb producing cells. HCPs in the cell culture fluid were recovered by centrifugation followed by 0.5/0.2 µm filtration. MabSelect SuRe and *N*-hydroxysuccinimide (NHS)-activated Sepharose 4 Fast Flow resins were obtained from GE Healthcare (Uppsala, Sweden). Coomassie Plus (Bradford) Protein Assay was from Thermo Scientific (Rockford, IL). Materials for SDS-PAGE were from Invitrogen/Life Technologies (Grand Island, NY). Dithiothreitol and iodoacetamide were from Sigma-Aldrich (St. Louis, MO) and trypsin was from Promega (Fitchburg, WI). Hydrophilic polypropylene AcropPrep 96 Filter Plates (0.45 µm) were from Pall Life Sciences (Ann Arbor, MI).

### Equipment

Protein A chromatography experiments were performed on an ÄKTA Explorer chromatographic system from GE Healthcare (Uppsala, Sweden). Analysis of peptide mixtures was carried out on a nano-ACQUITY UPLC® from Waters (Milford, MA) and LTQ or LTQ Velos mass spectrometers from Thermo Fisher Scientific (Waltham, MA). Immunoassays were performed using Gyros immunoassay platform from Gyros AB (Uppsala, Sweden).

### Methods

#### Immobilization of mAbs Through Cross-Linking to NHS-Activated Sepharose Resin

Purified mAb (20 mg) was immobilized to NHS-activated Sepharose 4 Fast Flow resin (1 mL) according to the manufacturer's instructions. Briefly, NHS resin was washed with 15 bead volumes of 1 mM hydrochloric acid prior to overnight incubation with mAb1, mAb2, mAb3, or mAb4, or incubation without a mAb (control) at 4°C. The mAb-Sepharose resin was then washed five times with 0.1 M sodium bicarbonate, 0.5 M sodium chloride at pH 8.3 to remove any excess of noncross-linked mAb. NHS groups were quenched by incubating the resin with 0.1 M Tris–HCl, pH 8.5 for 4 h. Three cycles of alternating high and low pH washes (0.1 M Tris–HCl, pH 8.5 and 0.1 M Sodium acetate, 0.5 M sodium chloride, pH 4.5) were performed after quenching. The mAb-Sepharose resin was then stored in phosphate buffered saline (PBS) at 4°C.

#### Capture of mAb-Associated HCPs Using mAb-Sepharose Resin

Six mg of HCPs from the clarified null CHO supernatant (quantity determined by ELISA assay) were added to 1 mL of the mAb-Sepharose resin and incubated in a spin column under mixing overnight at 4°C. The amounts of HCPs mixed with mAb were targeted to mimic the typical ratio of HCPs to mAb in the mAb-expressing cell culture supernatant (6 mg HCP/20 mg mAb = 300,000 ng HCP/mg mAb). Prior to elution of bound HCPs, the resin was washed six times with 1 bed volume (BV; 6 × 1 BV) of PBS to remove unbound HCPs. To elute HCPs associated with the mAb, 1 M guanidine hydrochloride at pH 4.5 (3 × 1 BVs) was applied to the resin. These elution conditions resulted in complete elution of bound HCPs as shown by the fact that subsequent elution with 2% sodium dodecyl sulfate (SDS) resulted in no additional elution of HCPs when analyzed using SDS-PAGE (data not shown).^17^ Each step of wash and elution was carried out under mixing for 10 min. HCPs in the elution pools were analyzed by ELISA for total HCP content and by SDS-PAGE for comparison of the HCP patterns of distribution obtained for each mAb evaluated. To identify the HCPs, each elution pool was treated with 10 mM dithiothreitol for 30 min at 37°C, followed by 50 mM iodoacetamide for 30 min at room temperature in the dark. Subsequently, the HCPs in the elution pools were digested overnight with trypsin at 37°C to generate peptides. The peptides were analyzed by mass spectrometry to identify the individual HCP species that associated with the mAbs.

#### Capture, Identification, and Quantification of HCPs Remaining Bound to mAb After Different Wash Conditions

A high throughput format of the experiment described above was conducted in 96-well filter plates to screen the effects of different wash modifiers on dissociating HCP–mAb interactions. All incubation, washing and elution steps were performed as described above with the exception of incorporation of a wash, containing the modifier(s) under evaluation, after the PBS wash step. Each step of wash and elution was carried out under shaking for 10 min. Total levels of HCPs in the elution fractions were measured by ELISA and the identity and relative levels of individual HCP species were assessed by mass spectrometry.

#### Protein A Chromatography

mAb1 CHO supernatant were loaded on MabSelect SuRe to 80% of the dynamic binding capacity at 10% breakthrough. The columns were re-equilibrated with five column volumes (CVs) PBS, washed with five CVs of either PBS as control wash or the other wash buffers and eluted with 50 mM sodium acetate pH 3.7. The levels of HCPs in the elution pools were assessed by ELISA.

#### SDS-PAGE Analysis of HCP–mAb Interaction Profiles

For SDS-PAGE, 10 µL of HCPs eluted from the mAb-Sepharose resins were loaded on 4–12% Tris–glycine gels and protein bands were stained with Coomassie blue (SimplyBlue Safe Stain) according to the manufacturer's instructions.

#### HCP ELISA

HCPs in all samples were quantified using a custom reagent set on the Gyros immunoassay platform. Briefly, reagents were prepared by immunizing sheep with a representative null CHO cell line harvest. The IgG antibodies against HCPs were purified from serum by affinity chromatography. The purified IgG antibodies were labeled with either biotin (Thermo Scientific, Rockford, IL) to capture antibodies onto plates or with Alexa647® (Life Technologies, Carlsbad, CA) for detection of HCPs on the Gyros platform. HCP samples were analyzed using the Gyros platform for nanoliter scale immunoassays in microfluidic channels according to the manufacturer's instructions. HCP concentrations were determined by regression to a standard curve made from the null CHO reagent. HCP values are reported in ng/mL.

#### Mass Spectrometry Analysis

Identification of HCPs was performed by separation of peptides on a nano-ACQUITY UPLC® system equipped with a 180 µm i.d. × 20 mm length C18 Symmetry trap column and a 100 µm × 100 mm C18 (Waters) reversed phase column operated at a flow rate of 400 nL/min (Buffer A: 0.1% formic acid; Buffer B: 0.1% formic acid in acetonitrile). Approximately 0.5 µg of each sample was injected onto the trap column in 1% Buffer B. Peptides were eluted from the column using a 330-min method for in-solution digestions. After the LC separation, the eluted peptides were analyzed online using a LTQ Velos (top five MS/MS method) mass spectrometer (Thermo Fisher Scientific) in data-dependent mode using collision-induced dissociation for MS/MS.

#### Data Analysis

The identity of each HCP present was determined using the Proteome Discoverer v. 1.3 software using the Sequest mode by searching mass spectral data against a CHO protein sequence database. Peptide quality scores were derived by processing against a decoy database using the Peptide Validator node within Proteome Discoverer that calculates the probability that the search algorithm incorrectly included a peptide in a sample. The false discovery rate (FDR), or the false positive rate, is a statistical value that estimates the number of false positive identifications among all identifications found by a peptide identification search. Peptides assessed with less than 5% FDR (medium and high confidence peptides) were retained, and those assessed with less than a 1% FDR (high confidence) threshold noted as high confidence. A minimum of two medium or high confidence (passing) peptides per protein were required to positively identify each HCP. The MS/MS spectra of peptides for those proteins which were identified by only one high confidence peptide were manually examined for coverage of three or more consecutive b- and y-ions and low number of abundant extraneous ions to determine their acceptance.

For calculating the relative quantities of individual HCPs, the spectral counting approach was utilized as previously described.^18^–^20^ Briefly, the spectral count (SpC) determined from the number of MS/MS spectra for each protein in each of the multiple LC-MS/MS datasets was divided by the molecular weight of each HCP identified in the elution pools to account for the effect of protein size on SpC. The relative mass of each HCP was calculated using the normalized spectral abundance factor (NSAF) as follows:


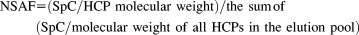



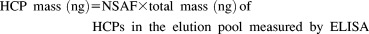


The relative amounts (ng) of HCPs in the elution pools from different wash conditions were expressed as percentage of HCP amounts recovered after PBS control wash and presented in Tables1–3.

The quantitative results presented in the tables are provided as an average of three experiments. By only including high abundant HCPs representing >1% of the total NSAF in each sample, more confident identification and quantification of HCPs is obtained due to significantly reducing variability of the levels of low abundant HCPs between experiments. Consistent data were obtained by ELISA thus further supporting the mass spectrometry results on the HCP levels in the samples.

## Results and Discussion

### Capture and identification of HCPs that associate with mAbs

**Figure 1:**
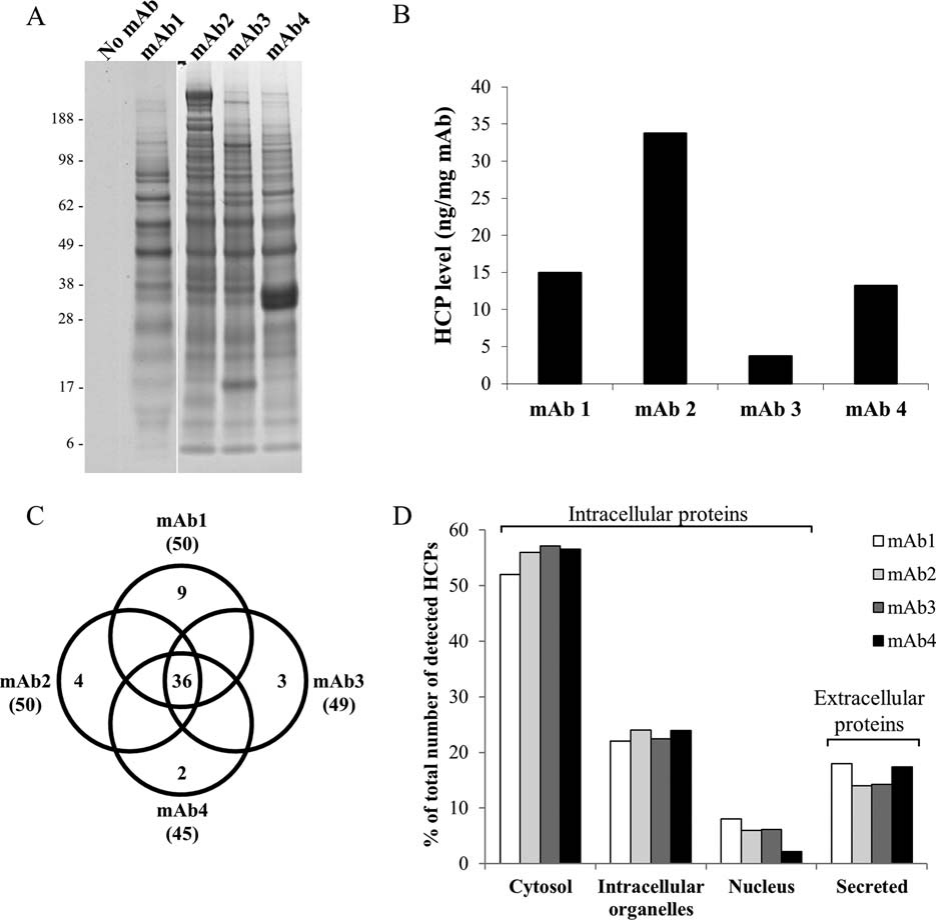
Characterization of HCP interactions with mAbs. (A) SDS-PAGE profiles of HCPs from the null CHO cell supernatant that bound to mAb1, mAb2, mAb3, and mAb4 compared to no mAb control. (B) The levels of HCP that bound to each mAb as measured by ELISA. (C) Venn diagram comparison of the numbers of HCP species that bound in common or uniquely to each mAb. Total numbers of HCPs identified for each mAb are in parentheses. (D) Subcellular distribution of HCPs that bound to each mAb. The number of HCP species in each category is expressed as percentage of the total number of HCPs identified.

A thorough proteomic analysis of HCPs that associated with different mAbs without interference from the mAb itself was achieved through employing immobilization of the mAb onto a resin surface by cross-linking. The immobilized mAb was allowed to interact with a pool of HCPs obtained from null-CHO cell supernatant. Sepharose-based resin was used to immobilize the mAb, as this resin was shown to have minimal to nondetectable levels of nonspecific HCP interactions, when used during Protein A capture step of mAbs.^8^,^12^ To confirm this, a control experiment was conducted in which the immobilization procedure was performed in the absence of the mAb. The Sepharose resin was incubated with the null-CHO cell supernatant. HCPs that were loosely adsorbed to the Sepharose resin were then washed off with PBS. To ensure efficient elution of tightly bound HCPs (if present), a stringent elution condition (guanidine hydrochloride) was used, and recovered HCPs were subsequently analyzed using SDS-PAGE as described in the Methods section. Indeed, no significant HCP bands were detected on the SDS-PAGE gel in the elution pool under the conditions used for this experiment (Figure 1A, “no mAb” lane).

To capture and recover HCPs that could bind to the mAb under typical operating conditions of Protein A chromatography in the manufacturing process, the same procedure described above for the control experiment was performed but in the presence of a purified mAb immobilized to the Sepharose resin. In the presence of mAb1, multiple HCP bands were observed on SDS-PAGE gel in the elution pools, indicating specific associations of HCPs with mAb1 (Figure 1A, “mAb1” lane). Further, examination of the gel band profiles of HCPs that interacted with mAb1, mAb2, mAb3, and mAb4 showed high similarities between the mAbs in many areas, in agreement with the high homology in the amino acid sequences of IgG molecules (Figure 1A). However, there were few distinct zones that were unique to each mAb, suggesting HCP interactions with the variable regions on the mAbs.

ELISA of the relative levels of recovered HCP that associated with each mAb showed that these mAbs have different HCP binding propensities (Figure 1B). For example, mAb2 bound a significantly higher total amount of HCP compared with mAb1, mAb3, and mAb4. These findings are consistent with studies in which spiking of different mAbs with the same HCP feedstock resulted in co-purification of different levels of HCPs when purified on Protein A chromatography.^9^,^16^,^21^

Identification of recovered HCP species that bound to each of the four mAbs was performed by subjecting the elution pools from the experiments described above to mass spectrometry analysis. Table1 lists the identified HCPs and their estimated masses. Only HCPs that represented >1% (as calculated in the Methods section) of the total mass of all detected HCPs for one or more of the mAbs is listed. Thirty-six HCPs were found to associate with all four mAbs, which is equivalent to 70–80% of the total number of HCPs that were identified to associate with each mAb (Figure 1C, shaded HCPs in Table1). This large number of HCPs found to bind to all four mAbs corroborates the high similarities of the HCP band profiles observed in Figure 1A. Consistent with a recent study in which a similar approach was used, a large number of these mAb-associated HCPs have also been found to associate with different mAbs.^16^ In addition, recent studies of HCPs in Protein A purified mAb products showed large overlap with HCPs identified in our study^22^,^23^ (highlighted in Table1). These results suggest that these HCPs likely bind to the shared constant regions of the mAbs. By similar reasoning, HCP species that bind uniquely to a single mAb likely associated with the variable domains of the mAbs. To examine whether any HCP impurities bind covalently to the mAb, a separate peptide mass fingerprinting analysis was performed to specifically searching for the covalently link with the mAbs included in this study. The result indicates no detectable covalent interactions of HCPs with the mAbs (data not shown).

**Table 1:** Comparison of Identified HCPs Associated With mAbs 1–4

Identified HCP	MW	pI	HCP Mass (ng/20 mg mAb)			
			mAb1	mAb2	mAb3	mAb4
14-3-3 protein epsilon	7.89	6.52	9.0	24.3	ND	ND
40S ribosomal protein S15a	14.83	10.13	9.0	ND	ND	ND
40S ribosomal protein S17	15.51	9.85	ND	20.3	ND	10.3
40S ribosomal protein S20	15.21	9.94	ND	20.3	2.3	8.7
40S ribosomal protein S21	9.14	8.32	9.0	ND	ND	ND
40S ribosomal protein SA	19.72	9.29	18.0	28.4	2.3	10.9
60S acidic ribosomal protein P0*	29.87	8.51	9.0	20.3	2.3	7.9
60S acidic ribosomal protein P2	11.67	4.54	24.2	26.3	3.0	13.5
60S ribosomal protein L12	14.69	9.55	12.6	20.3	2.3	10.3
60S ribosomal protein L23a	23.11	10.45	ND	ND	2.3	10.3
78 kDa glucose-regulated protein†‡	72.33	5.16	9.0	26.3	3.0	10.3
Acid trehalase-like protein 1	29.46	5.62	ND	ND	ND	9.5
Actin, alpha cardiac muscle 1†‡	41.99	5.39	9.0	20.3	2.3	7.9
actin, cytoplasmic 1†‡	41.71	5.39	13.4	20.3	3.3	7.9
Alpha-enolase†‡	46.67	6.16	12.6	46.6	5.4	34.8
Annexin A2	27.02	5.97	10.8	ND	ND	ND
Aspartyl aminopeptidase	51.91	7.12	ND	ND	2.3	ND
Biglycan	41.61	7.31	19.7	20.3	2.3	9.5
**Cathepsin B**	37.48	6.13	9.0	24.3	2.5	11.9
Clusterin†‡	51.72	5.74	12.6	20.3	2.3	7.9
Cofilin-1†	18.52	8.09	22.4	24.3	3.6	11.1
Cullin-associated NEDD8-dissociated protein 1	133.54	5.74	9.0	28.4	2.3	7.9
Elongation factor 2†	95.26	6.83	9.0	20.3	2.3	7.9
Eukaryotic translation initiation factor 2 subunit 1	36.13	5.14	ND	22.3	3.8	17.4
Galectin-3	32.42	7.37	ND	ND	ND	15.8
Glutathione S-transferase P 1*	23.62	7.80	9.0	26.3	4.5	15.8
Glyceraldehyde-3-phosphate dehydrogenase†	35.73	8.34	16.1	40.5	4.3	18.2
GTP-binding nuclear protein Ran	24.47	8.34	12.6	20.3	2.3	ND
Guanine nucleotide-binding protein beta-2-like 1	30.44	7.39	9.0	20.3	ND	ND
Heat shock cognate 71 kDa protein†,‡	70.76	5.36	9.0	22.3	2.3	7.9
Histidine-tRNA ligase	57.39	6.95	ND	20.3	ND	ND
Histidyl-tRNA synthetase, cytoplasmic	40.67	5.08	ND	20.3	ND	ND
Histone H2A type 1	28.45	11.03	15.2	ND	ND	ND
Histone H2A type 4	14.24	11.02	ND	30.4	2.3	ND
Histone H2B type 1-N	13.93	10.32	15.2	ND	2.3	ND
Lactadherin	16.14	9.41	28.7	34.4	3.2	14.3
L-lactate dehydrogenase A chain	36.50	7.42	9.0	ND	ND	ND
Phosphotyrosine protein phosphatase	17.90	6.01	ND	20.3	2.3	8.7
**Matrix metalloproteinase-19**	58.90	7.88	9.0	ND	ND	ND
Metalloproteinase inhibitor 1‡	22.39	8.47	9.0	20.3	2.3	7.9
Nidogen-1†,‡	30.07	8.07	10.8	ND	ND	ND
Nucleoside diphosphate kinase A	17.18	6.33	ND	ND	7.9	ND
Nucleoside diphosphate kinase B	17.33	7.99	ND	ND	4.3	ND
Out at first protein-like	17.72	8.06	9.0	20.3	3.0	7.9
Peptidyl-prolyl cis-trans isomerase A *	17.89	8.28	9.0	20.3	2.3	7.9
Peroxiredoxin-1†‡	22.25	8.05	27.8	46.6	4.3	19.8
Phosphoglycerate mutase 1*	20.19	8.12	9.8	ND	ND	ND
Plectin-1	83.37	8.54	ND	40.5	ND	ND
Proteasome subunit alpha type-7	23.76	8.48	ND	ND	2.3	7.9
**Protein disulfide-isomerase A6**	28.38	4.91	9.0	46.6	3.8	15.8
Protein S100-A6	10.00	5.48	9.0	ND	ND	ND
Putative phospholipase B-like 2	65.50	6.28	9.0	36.5	3.0	ND
Pyruvate kinase isozymes M1/M2†,‡	51.53	7.68	29.6	54.7	5.6	23.8
Septin-2	37.05	6.18	9.0	20.3	2.3	7.9
**Serine protease HTRA1**†,‡	28.70	7.03	9.0	20.3	2.3	7.9
Serpin H1	46.53	8.68	9.0	20.3	2.3	7.9
Sulfated glycoprotein 1†,‡	27.36	5.49	9.0	38.5	3.8	9.5
T-complex protein 1 subunit alpha†,‡	60.30	5.99	14.4	24.3	2.5	9.5
T-complex protein 1 subunit beta†,‡	57.45	6.46	16.1	36.5	3.6	15.8
T-complex protein 1 subunit delta†,‡	42.11	8.27	9.8	20.3	2.3	7.9
T-complex protein 1 subunit eta†,‡	54.87	7.42	9.8	20.3	2.3	7.9
T-complex protein 1 subunit gamma†,‡	60.58	6.64	13.5	28.4	2.5	10.3
T-complex protein 1 subunit theta†	22.21	4.91	20.6	30.4	4.5	12.7
T-complex protein 1 subunit zeta†,‡	57.94	6.90	11.7	20.3	2.5	7.9
Triosephosphate isomerase	16.42	8.19	ND	30.4	4.5	15.8
Vimentin‡	44.50	4.81	18.0	24.3	2.3	10.3
V-type proton ATPase subunit C 1‡	43.89	7.46	ND	20.3	ND	ND

Gray-shaded HCPs bound to all four mAbs. HCPs of potential risk to cause enzymatic degradation of the mAbs are indicated in bold text. ND indicates no detection of the HCPs or levels below 1% of the total HCP mass as calculated in Methods section.

* HCPs previously found in Protein A-purified mAb product by Zhang et al.^23^

† HCPs previously found in Protein A-purified mAb product by Doneanu et al.^22^

‡ HCPs associated with other mAbs identified in Levy et al.^16^

Some of the HCPs identified could be considered of high risk, as they may have a potential impact on mAb structural stability due to their established functions (e.g., enzymatic), as described in the literature. These HCPs are cathepsin B, matrix metalloproteinase-19, protein disulfide isomerase (PDI), and serine protease HTRA1 (Table1). Except for matrix metalloproteinase-19, which bound only to mAb1, these HCPs bound to all four mAbs. While cathepsin B, matrix metalloproteinase-19, and serine protease HTRA1 are proteolytic enzymes that could potentially cause fragmentation of the mAb molecules,^5^ PDI could potentially cause reduction of the disulfide bonds in mAbs, as has been demonstrated for disulfide bonds in insulin.^24^ In addition, to assess the potential impact of the identified HCPs on mAb stability, it is possible that *in silico* tools could also be used for prediction of immunogenicity of each HCP to identify HCPs with other potential risk, which could impact patient safety.^1^

To further characterize the HCPs that bound to each mAb, UniProtKB database (ExPASy Bioinformatics Resource Portal, http://www.expasy.org) was used to investigate the subcellular localization of the identified HCPs. As expected, the majority of the HCPs that bound to the four mAbs were intracellular proteins (e.g., cytosol, intracellular organelles, and nucleus; Figure 1D). These HCPs were likely released to the null supernatant due to low cell viability and breakage of cells during the harvest process (cell separation by centrifugation). HCPs that are naturally secreted to the supernatant during the cell culture process represented only ∼15% of the HCPs that bound to the mAbs (Figure 1D). Thus, higher cell viability and gentle harvest process could decrease the levels of intracellular HCPs that interact with the mAb and be carried over during the purification process and could potentially affect HCP distribution in the mAb product. Similar subcellular distribution of HCPs that bound to each mAb was observed except for mAb4, which bound to slightly less nuclear HCPs compared with the other three mAbs (Figure 1D). However, these differences were small, which reflects the high similarity in structural properties of the IgG molecules.

Although we did not perform studies to show why a specific HCP bound to a certain mAb, we assessed the reason for HCP interactions with the mAb through examination of the functional properties of different HCPs in the literature and correlating those with known structural features of the mAbs. For example, the HCPs acid trehalase-like protein 1 and galectin-3 were found to bind exclusively to the IgG2 mAb4 but not to the IgG1 molecules mAb1, mAb2, or mAb3 (Table1). These two HCPs are known to be carbohydrate-binding proteins.^25^,^26^ Therefore, this preferential binding to mAb4 observed may be due to the higher glycosylation levels as well as the specific glycosylation pattern of IgG2 compared to IgG1.

### Dissociation of HCP–mAb interactions using wash modifiers

To enhance our understanding on clearance of HCPs that associate with the mAb, the approach of immobilizing the mAb onto the resin described in the current work was employed to monitor the effects of different wash modifiers on dissociating individual HCP–mAb interactions. A thorough screening of wash modifiers was conducted to determine optimal conditions to dissociate HCP–mAb1 interactions. The optimized wash conditions for mAb1 were then tested to dissociate HCP interactions with mAb2, mAb3, and mAb4. Accordingly, a set of experiments was conducted in a High-throughput format, in which mAb1-Sepharose resin was incubated with null CHO supernatant, re-equilibrated with PBS, and then washed with each of the wash modifiers being studied. Bound HCPs whose associations with the mAb were not broken by the wash were eluted using guanidine hydrochloride. The total HCP levels in the elution pools after each wash were assessed using ELISA.

**Figure 2:**
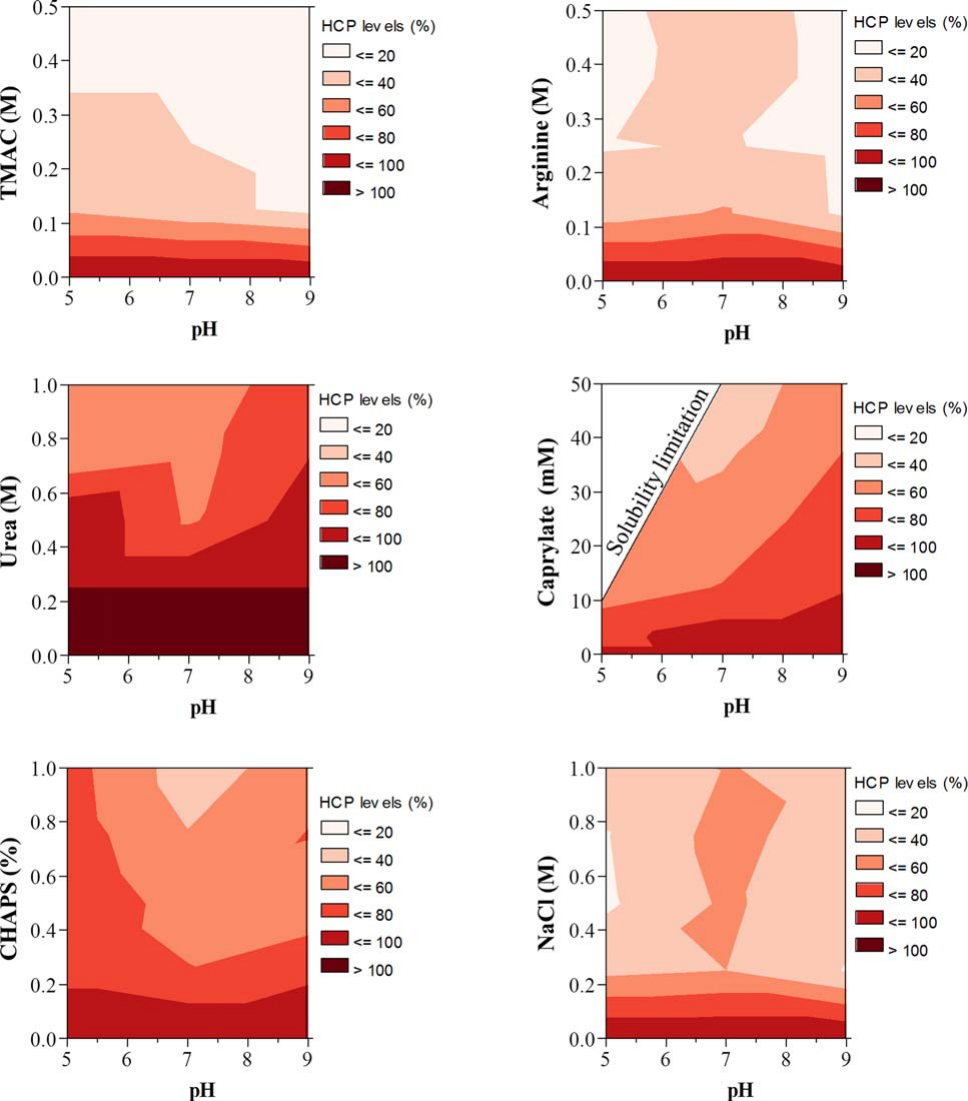
Effects of wash modifiers on removal of mAb1-associated HCPs. The level of HCPs remaining after each wash condition was assessed by ELISA and expressed as a percentage of the level obtained after wash with no added wash modifier.

The total levels of HCPs that remained bound to mAb1 after different wash conditions are shown in Figure 2. As expected, increasing the concentration of each wash modifier reduced the levels of HCPs remaining bound to mAb1. For each wash modifier, clearance of HCPs appeared to be independent of pH at low modifier concentrations (Figure 2). Washes with tetramethylammonium chloride (TMAC) or arginine appeared to be the most effective modifiers to break HCP–mAb1 interactions and reduce the levels of HCPs to ∼20% of control (Figure 2). Moderate HCP reduction (∼40–60% of control) was observed for the modifiers urea, sodium caprylate, CHAPS, and sodium chloride. Both TMAC and arginine have recently been shown to improve HCP clearance when used as post load wash during Protein A affinity chromatography.^27^,^28^ Our results suggest that the mechanism of this improvement in HCP clearance could be through the effects of these two modifiers on dissociating HCP–mAb interactions. While the mechanism of TMAC to disrupt protein–protein interactions is not well established, the effects of arginine on modulating protein retention in ion exchange, hydrophobic interaction chromatography has been described in literature by Arakawaa et al.^29^,^30^ In addition to electrostatic and hydrophobic interactions, the guanidium moiety on arginine has been shown to affect hydrogen bonding which can lead to further disruption of protein–protein interactions.^31^

**Figure 3:**
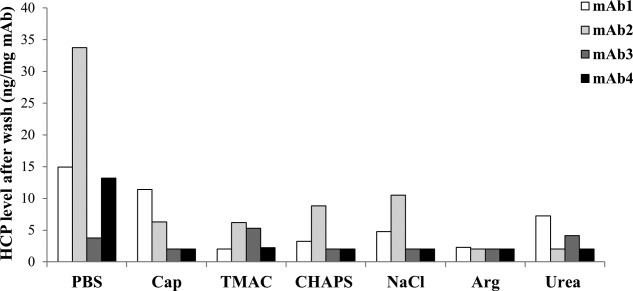
Effects of wash modifiers on removal of HCPs associated with different mAbs. HCP levels were assessed by ELISA after wash with phosphate buffer (PBS), 50 mM sodium caprylate (Cap), 0.5 M tetramethylammonium chloride (TMAC), 1% (w/v) CHAPS, 1 M sodium chloride (NaCl), 0.5 M arginine-HCl (Arg), and 1 M urea. All wash modifiers were prepared in phosphate buffer at pH 7

To test whether the optimized wash conditions for dissociating HCP–mAb1 interactions are also effective to dissociate the interactions with other mAbs, experiments were conducted in which mAb2, mAb3, and mAb4 were washed with the same wash buffers (PBS was used as a control wash). The effectiveness of a particular wash on breaking HCP–mAb interactions appeared to be mAb dependent for all wash modifiers evaluated, except for arginine which showed consistent clearance of HCPs associated with all four mAbs (Figure 3). As was demonstrated in Figure 1, there were diversities in both the populations and the levels of HCPs associated with each mAb. This could potentially account for the differences observed in the effects of the wash modifiers to break HCP interactions with different mAbs.

**Figure 4:**
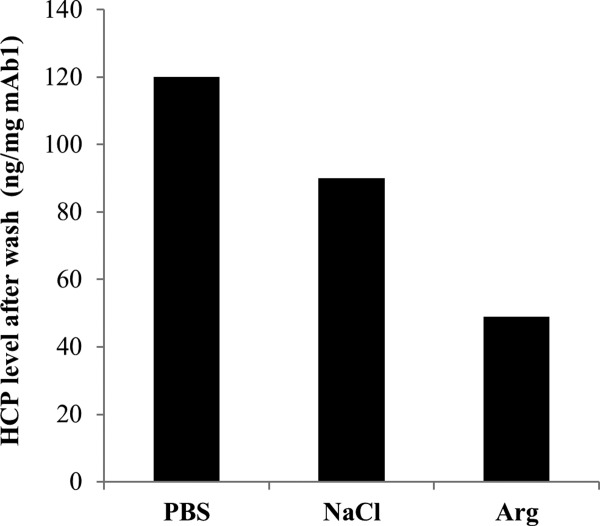
Validation of the effectiveness of wash modifiers on HCP removal during purification of mAb1 on conventional Protein A chromatography. Wash modifiers were prepared in phosphate buffer at pH 7 at the indicated concentrations. The levels of HCPs in the Protein A elution pools were measured by ELISA.

To validate the effectiveness of the wash modifiers on dissociating HCP–mAb1 interactions on a conventional Protein A unit operation, HCP levels of MabSelect SuRe elution products were assessed with different modifiers. Following wash with NaCl or arginine, the reduction in HCP levels co-eluted with mAb1 on Protein A appeared to follow similar trends to those observed using the mAb immobilization approach (Figures 3 and 4). No significant effects on mAb1 yield were observed compared with PBS control wash (data not shown).

### Monitoring clearance of individual HCPs that interact with the mAb by mass spectrometry

In addition to using ELISA to evaluate clearance of HCPs, mass spectrometry was used to identify individual HCPs that remained bound to mAb1-Sepharose after each wash and to assess their levels. Table2 shows a heat map of the levels of 20 of the identified HCPs after washes with different modifiers. The levels of each HCP are expressed as percentage of HCP masses remaining after a PBS control wash as described in Methods section. In agreement with the ELISA results (Figure 2), the relative quantification of HCPs by mass spectrometry showed that washing mAb1 with TMAC or arginine appeared to be the most effective wash conditions for breaking individual interactions between the HCPs and mAb1 (Table2). While both TMAC and arginine markedly decreased the levels of HCPs, the levels of some individual HCPs after wash with sodium caprylate, urea, sodium chloride, or CHAPS remained relatively high (Table2). Moreover, the levels of high-risk HCPs that associated with mAb1 such as cathepsin B, serine protease HTRA1, and PDI A6, were significantly reduced after wash with TMAC or arginine.

**Table 2:** Mass Spectrometric Identification and Relative Quantification of HCPs That Remained Associated With mAb1-Sepharose After Wash With Different Modifiers

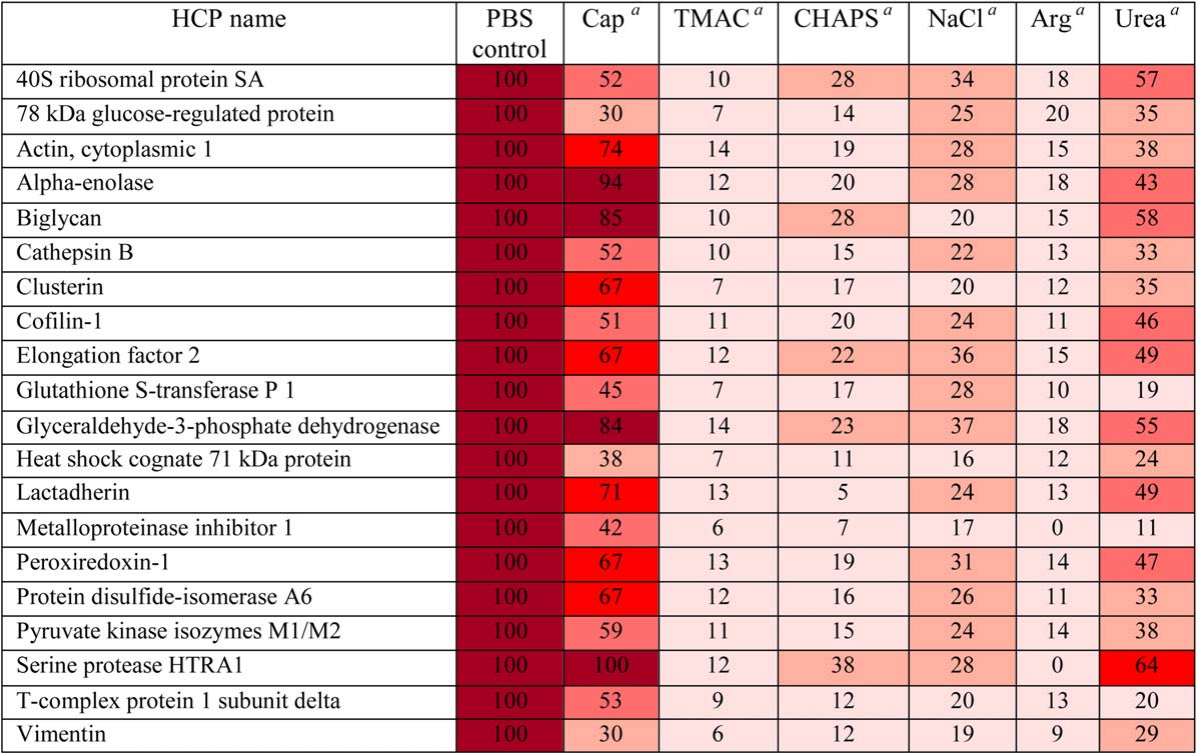

The levels of HCPs are expressed as percentage of levels observed for PBS control. 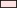 0-20 %, 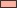 21-40 %, 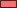 41-60 %, 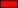 61-80 %, 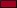 81-100 %

Cap: 50 mM sodium caprylate; TMAC: 0.5 M tetramethylammonium chloride; CHAPS: 1% (w/v) CHAPS; NaCl: 1 M sodium chloride; Arg: 0.5 M arginine–HCl; and urea: 1 M urea. All wash modifiers were prepared in phosphate buffer at pH 7.

**Figure 5:**
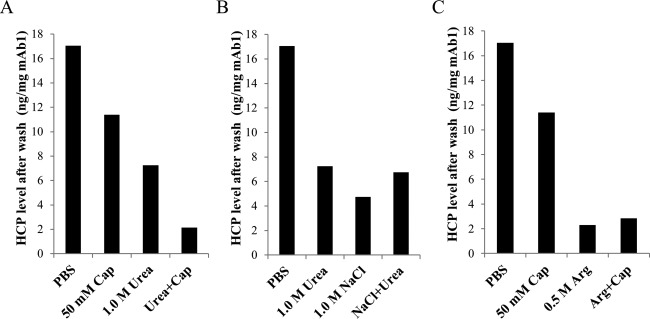
Effects of combinations of wash modifiers on removal of mAb1-associated HCPs. All wash modifiers were prepared at the indicated concentrations in phosphate buffer at pH 7. For combined modifiers, the same concentrations were used as for the single modifiers using the same buffer system. The levels of HCPs remaining after each wash condition were measured by ELISA.

**Figure 6:**
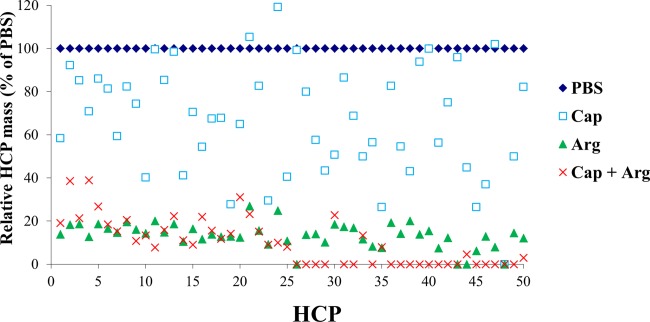
Effects of combinations of wash modifiers on breaking interactions of individual HCP with mAb1. The levels of individual HCPs remaining after each indicated wash were obtained from mass spectrometry analysis and are expressed as a percentage of the levels after PBS control wash as described in Method section. The *x*-axis shows individual HCP species.

The effects of combining two wash modifiers on dissociating HCP–mAb1 interactions were assessed by both ELISA and mass spectrometry to determine the total and individual HCP levels, respectively, after each wash combination. A combination of two wash modifiers could potentially improve HCP clearance through synergic effects in mitigating different types of protein–protein interactions such as electrostatic, hydrogen bond, and hydrophobic interactions. Indeed, combination of urea and sodium caprylate in the wash significantly improved HCP removal as indicated by the reduction in both the total HCP levels measured by ELISA (Figure 5A) and the levels of individual HCPs that remained associated with mAb1 (Table3). However, when the two modifiers urea and sodium chloride were combined in the wash, no further reduction of HCP levels was observed as determined by ELISA and mass spectrometry analysis of the total and individual HCP levels, respectively (Figure 5B and Table3). In another study, a combination of sodium caprylate and arginine appeared to be as beneficial as a wash with arginine alone as indicated by comparable HCP levels remaining after these different wash conditions using ELISA assay (Figure 5C). However, assessment of the levels of individual HCPs by mass spectrometry analysis showed that combining caprylate with arginine reduced the levels of a large number of HCPs down to undetectable levels (0%) compared with HCP levels after the arginine wash alone (Figure 6). These results show that our approach enables identification of HCPs as well as monitoring the removal of individual HCP after a particular wash condition.

**Table 3:** Comparison of the Levels of HCPs That Remained Associated With mAb1-Sepharose After Wash With One or Combination of Two Modifiers

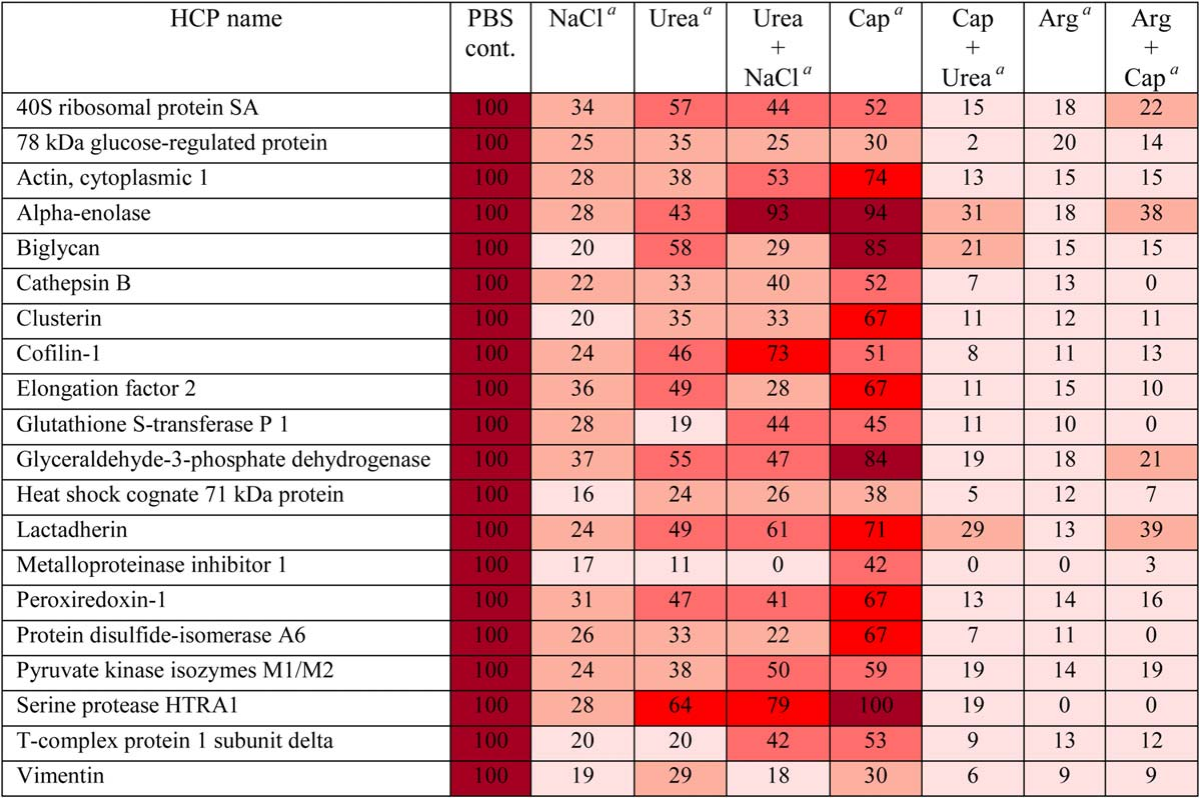

The levels of HCPs are expressed as percentage of levels observed for PBS control. 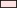 0-20 %, 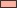 21-40 %, 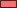 41-60 %, 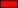 61-80 %, 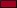 81-100 %

NaCl: 1 M sodium chloride; urea: 1 M urea; cap: 50 mM sodium caprylate; arg: 0.5 M arginine–HCl. The same concentrations were used for combination of two wash modifiers as for single modifiers. All wash modifiers were prepared in phosphate buffer at pH 7.

These results show that, in addition to monitoring the total HCP clearance by ELISA, it is useful to utilize our approach to rapidly identify individual HCPs with enzymatic function that could be responsible for degradation events of the mAb molecule. This could enable selection of optimal wash conditions targeted to remove these high-risk HCPs during Protein A mAb capture step.

## Conclusions

This work demonstrates an approach to recover and identify HCPs that associate with the mAb as well as study the effects of different wash conditions on dissociating individual HCP–mAb interactions. This approach could potentially facilitate rapid development of wash conditions to improve HCP clearance during the Protein A capture step for mAb manufacturing. Furthermore, identification and monitoring of the levels of individual HCPs can enable rational assessment of potential risks posed by individual HCPs on the mAb stability and thus enable rational selection of wash conditions to optimize removal of such HCPs. In addition, identification of HCPs could enable assessment of potential safety risks by using *in silico* or experimentally validated prediction tools to predict the immunogenicity potential of each identified HCP. Our work provides empirical understanding of HCP association profiles with the mAb for different wash conditions. In contrast to an ELISA method that only provides quantification of the total level of HCPs, our approach not only provides information about the identity of HCPs, but also their relative levels remaining after different wash conditions. This study shows that, even if different wash conditions result in comparable total HCP levels or improvement in HCP clearance, the composition of the HCP population as well as the levels of individual HCPs that remain associated with the mAb can be quite different for each condition. This is especially important if some high-risk HCPs are removed using one wash condition as opposed to another.

In conclusion, the approach described here can be utilized to support targeted selection of wash conditions for Protein A chromatography aiming to not only reduce the total levels of HCPs but also effectively reduce the levels of individual HCPs that may have potential high risks. Moreover, this approach can be used to identify high-risk HCPs that associate with the mAb and are difficult to remove so that other options such as targeted gene knockdown in the cell line could be considered to eliminate or reduce the levels of these HCPs.
